# A rare case of group A streptococcal toxic‐shock syndrome in a postpartum adolescent leading to multi‐organ failure

**DOI:** 10.1002/ccr3.2799

**Published:** 2020-03-16

**Authors:** Samuel Cortez Granados, Jonathan Batsch, Asona Lui, Jacob Hessman, Nika Gloyeske, Apurva Panchal

**Affiliations:** ^1^ Department of Pediatrics University of Kansas Medical Center Kansas City KS USA; ^2^ Department of Pathology and Laboratory Medicine University of Kansas Medical Center Kansas City KS USA; ^3^ Department of Pediatric Critical Care Medicine University of Kansas Medical Center Kansas City KS USA

**Keywords:** hysterectomy, intravenous immunoglobulin, pregnancy, teenager, toxic‐shock syndrome

## Abstract

Teenage pregnancy is not uncommon, but given the age of the patient, experience, and competency among medical providers varies. While toxic‐shock syndrome from group A streptococcus is rare in teenage pregnancy, observed is a gap in care of bridging.

## Graphical abstract

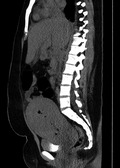

## 1 INTRODUCTION

Teenage pregnancy is not uncommon, but given the age of the patient, experience, and competency among medical providers varies. Here, we describe a 15‐year‐old female on postpartum day 3 who was admitted to the pediatric ICU with fever and hemodynamic instability from septic shock. Upon admission, the patient required intubation, vasoactive agents, and antibiotics. An emergent hysterectomy was done, and cultures grew group A streptococcus (GAS). On day 3 of admission, she developed subsequent multi‐organ failure. This rare complication may be so fulminant that the diagnosis is often made too late to provide lifesaving treatment. GAS toxic‐shock syndrome (TSS) should always be part of the differential diagnosis when a pregnant or postpartum patient presents with septic shock. While this is a rare occurrence in teenage pregnancy, observed is a gap in care of bridging adolescents into adult medicine, especially in obstetrics and postpartum care.

Toxic‐shock syndrome is a rare, but life‐threatening illness characterized by multi‐organ failure. The known causes of TSS are *Staphylococcus aureus* and group A streptococcus. These bacteria release super antigens that cause massive release of inflammatory mediators leading to capillary leak, shock, and tissue damage. In a recent study, pregnant and postpartum women were shown to have up to a 20‐fold increase risk of developing TSS.^1^ The annual US incidence of GAS infection in postpartum women is 5 per 100 000.^2^ Of these women, only a small percent will develop TSS and sequential organ failure requiring immediate intervention. When TSS from GAS develops within four days of delivery, the mortality rate approaches 60%.^3^ Here, we report a case of a 15‐year‐old girl who developed GAS TSS 3 days postpartum, complicated by multi‐organ failure and required emergent hysterectomy. To the best of our knowledge, this is the first case report of an adolescent developing postpartum GAS TSS.

## 2 CASE PRESENTATION

A 15‐year‐old G1P0 Caucasian female with a past medical history of well‐controlled asthma and esophageal reflux recently delivered a baby girl vaginally at 39‐week and 3‐day gestational age. Her pregnancy was complicated by tobacco use, preeclampsia, and urinary tract infection at time of delivery due to GAS. Prenatal testing for infectious etiologies was unremarkable, including human papilloma virus (HPV), human immunodeficiency virus (HIV), rapid plasma reagin (RPR) for syphilis, and group B streptococci (GBS). Per the rural hospital, foul smelling, meconium‐tinged amniotic fluid was noted at the time of delivery, followed by vaginal placental extraction. Mother's vital signs were within normal limits. The baby was febrile at birth with a temperature of 101.6°F (38.6°C) and was transferred to the neonatal ICU for broad‐spectrum IV antibiotics. The baby's course was noncomplicated. On postpartum day 3, the mother developed fever, acute onset abdominal and chest pain, tachycardia, and significant hypotension. She was diagnosed with septic shock and was transferred to our pediatric ICU.

Upon presentation, she was nonresponsive to verbal commands, febrile, and had a heart rate of 144 beats/min, blood pressure of 85/42 mm Hg, and respiratory rate of 23 breaths/min. Aggressive fluid resuscitation with a 2 L isotonic fluid was ineffective, and she was started on a continuous norepinephrine 0.15 mcg/kg/min infusion. Subsequent hypoxemia led to intubation, and she was placed on invasive positive pressure ventilation shortly after arrival. Phenylephrine 150 mcg/kg/min and vasopressin 0.4 units/h were added to optimize perfusion. Initial laboratory studies revealed mild leukocytosis with WBC of 12.7 K/UL, hemoglobin of 8.4 g/dL, platelet count of 57 000/UL, lactate of 6.6 mmol/L, severely elevated CRP of 39.51 mg/dL and procalcitonin of 11.6 ng/mL, INR of 1.6 (normal = 0.8‐1.2), and a significantly elevated D‐dimer of 99 000 ng/mL (normal value <50 years old is <500 ng/mL). She also had profound metabolic acidosis. Clinical picture and laboratories were consistent with septic shock and secondary disseminated intravascular coagulopathy. She was started on clindamycin, vancomycin, and zosyn for broad‐spectrum coverage. The patient received a unit of blood and fresh frozen plasma. Obstetrics and Gynecology (OBGYN) was consulted and determined that her clinical picture was most consistent with endometritis.

**Figure 1:**
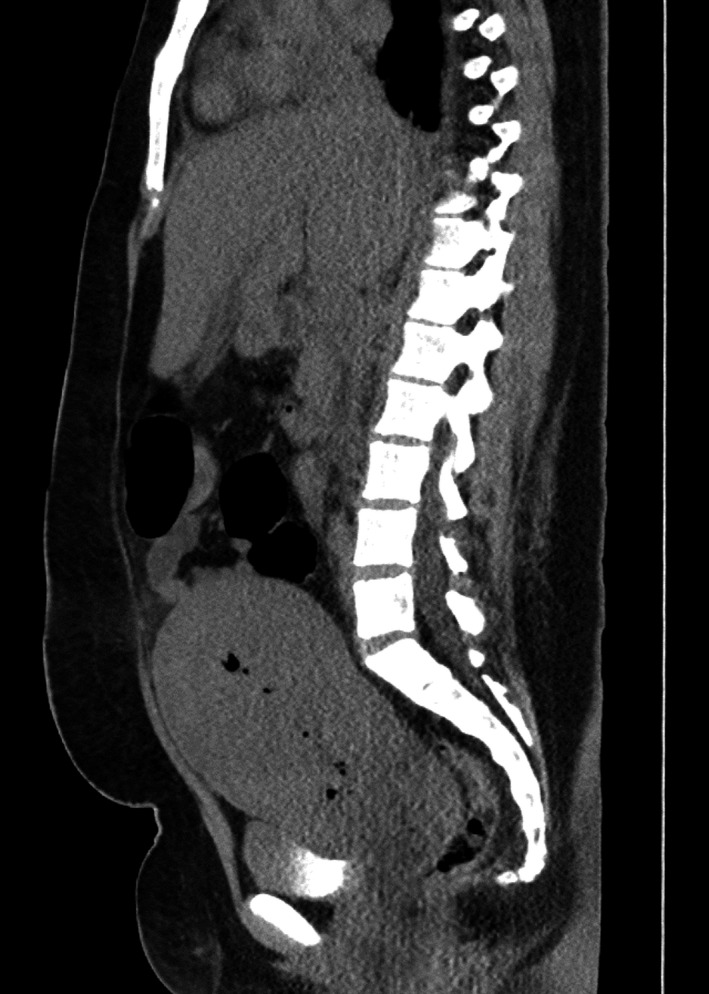
CT abdomen and pelvis, sagittal view. HMG (28 cm), SMG (17.5 cm), hepatic steatosis, small/trace amounts of ascites, enlarged uterus, and tiny pockets of air in the endometrial canal

**Figure 2:**
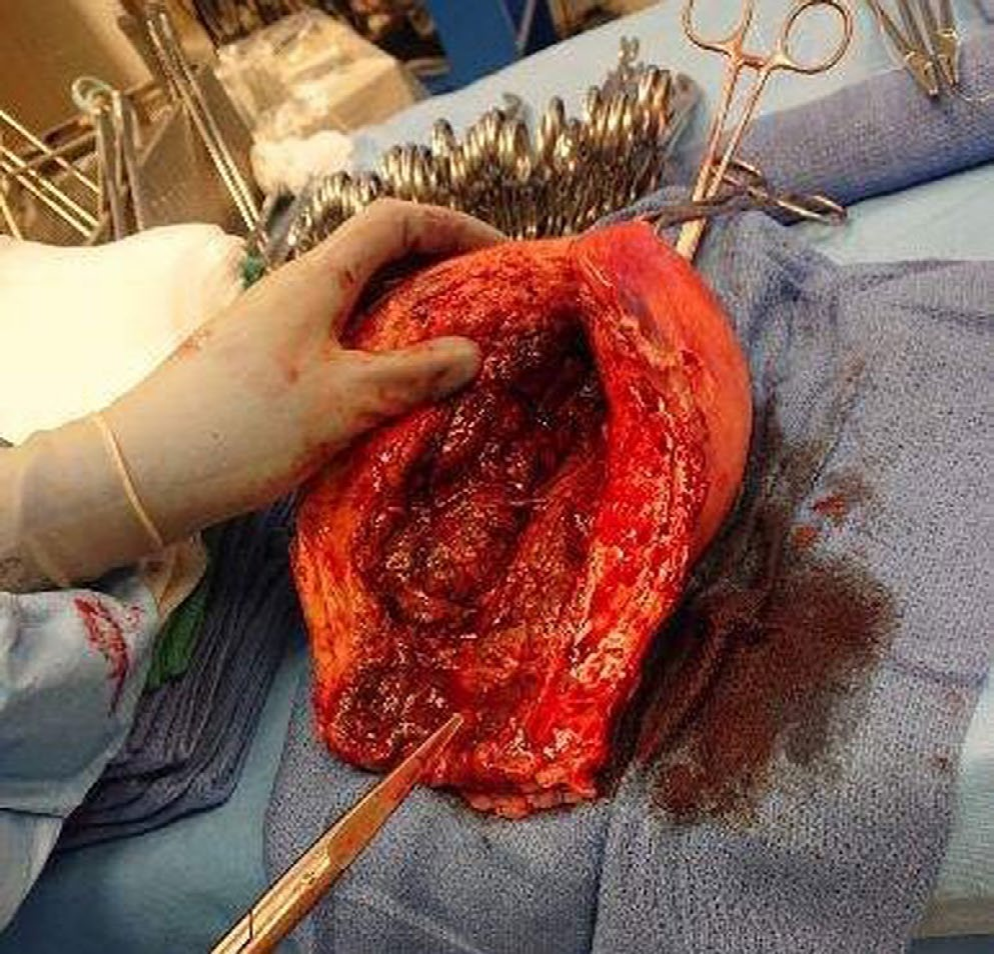
Gross examination of the uterus upon removal. Examination is consistent with the size of a postpartum uterus. Marked necrotic changes in the endometrium can be visualized, along with disruption of normal uterine architecture, all consistent with GAS endometritis

**Figure 3:**
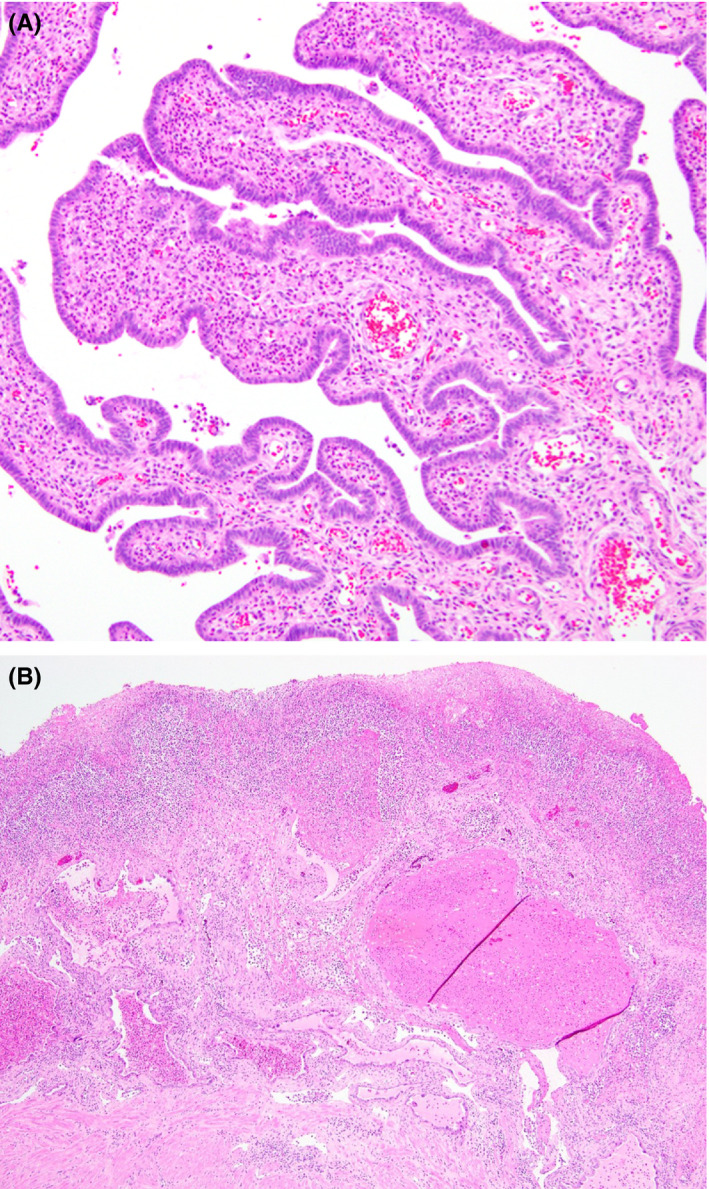
A, Fallopian tubes. Acute inflammatory infiltration into the fallopian tube plicae (H&E, 200×). B, Uterus. Marked acute and chronic inflammation intermixed with fibrin and necrosis of the endometrial lining overlying rare preserved endometrial glands with hemorrhage and pregnancy‐related changes in the left lower corner (H&E, 100×)

CT imaging was done and confirmed the diagnosis of endometritis with enlarged uterus. Within 24 hours upon arrival, she was taken to the operative room for exploratory laparotomy as her clinical condition continued deteriorating. A grossly edematous uterus (Figure 1) with massive necrosis, more than 75% of endometrium, was observed. It was decided at that time to perform a total abdominal hysterectomy with salpingectomy. Uterine tissue was sent for cultures, which along with blood and urine cultures came back positive for group A streptococci. Her antibiotics were subsequently narrowed to a 14‐day course of IV clindamycin and penicillin in accordance with GAS endometritis guidelines. Final surgical pathology revealed an enlarged, friable endometrium with significant necrosis and ischemia (Figures 2 and 3) consistent with septic endometritis.

**Figure 4:**
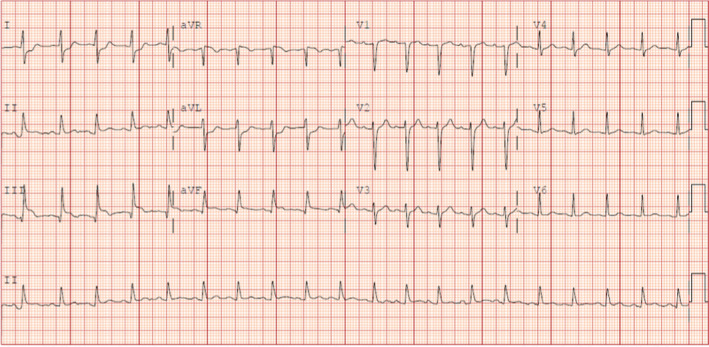
EKG with sinus tachycardia and inferior limb ST segment elevation and lateral limb lead ST segment depression

On day 3 of admission, she developed multi‐organ failure including acute respiratory distress syndrome and cardiovascular dysfunction leading to cardiogenic shock. The markers of cardiac injury were elevated with B‐type natriuretic peptide (BNP) reaching a peak of 1724 pg/mL (normal value is 0‐100 pg/mL) and troponin I reaching levels of 49.3 ng/mL (normal value 0.0‐0.05 ng/mL). Electrocardiography showed diffuse ST elevations (Figure 4). Echocardiogram revealed cardiomyopathy with severely diminished cardiac functions and a left ventricular ejection fraction of 25%. Due to concerns for coronary artery dissection, the patient was taken for cardiac catheterization, which was normal. All vasoactive agents were weaned down as tolerated, and she was placed on an intravenous milrinone infusion at 0.5 mcg/kg/min for her diminished cardiac function. She was started on intravenous hydrocortisone as we suspected her cardiomyopathy was likely the result of significant systemic inflammation. In addition to large dose steroid, five doses of 1 g of intravenous immunoglobulin (IVIG) were given to help control the systemic spread of inflammatory processes.

Over the course of two weeks, her cardiac function improved with milrinone, hydrocortisone, and IVIG. She was gradually weaned off all vasopressors (Table S1 for timeline and dosage of each vasopressor), and her cardiac functions and tissue perfusions stabilized. BNP, troponin I, lactate, CRP, and D‐dimer slowly normalized. She was eventually extubated to high‐flow nasal cannula and then slowly to room air. Her milrinone was eventually switched to lisinopril and carvedilol. She completed her fourteen‐day course of clindamycin and penicillin G. Her last echocardiogram prior to discharge demonstrated a normal LVEF of 73%. After 17 days of hospitalization, she was discharged in stable condition with outpatient follow‐up in pediatric cardiology and OBGYN.

## 3 DISCUSSION

Puerperal infections cause morbidity in 5%‐10% of all pregnant women with over 75 000 deaths globally each year.^4^ Most deaths occur in countries with poor prenatal care. Sepsis during pregnancy is infrequent. Less than 1% of pregnant women get bacteremia, and of these, only a handful develops sepsis and septic shock (0.01% of all deliveries).^2^ Nevertheless, they remain among leading causes of pregnancy‐associated death worldwide. GAS infection is one of the fatal infections that occurs during pregnancy and the postpartum period. Severe sepsis from GAS still carries a substantial amount of maternal mortality (40%‐60%) in developed countries.^3^ The diagnosis of streptococcal toxic‐shock syndrome (STSS) criteria was first published in 1993 and includes the isolation of group A streptococcus (GAS) from a sterile or nonsterile site associated with clinical signs of severity. Findings of GAS in postpartum women can be nonspecific which results in delayed treatment. By the time the diagnosis of GAS TSS is made, the disease can be advanced and involve multiple organs, as in this case. The management can be divided into supportive care, early targeted antibacterial therapy (including intravenous clindamycin), surgical removal of infected nidus, and potential use of IVIG.

GAS is a gram‐positive, beta‐hemolytic streptococcus. It colonizes human skin and mucus membranes but rarely resides in the vagina. The vaginal colonization rate of GAS in low‐risk pregnancies is 0.03%.^5^ This low prevalence of colonization is why it is not normally screened for during routine prenatal care. GAS causes a wide array of disease including cellulitis, pharyngitis, necrotizing fasciitis, and toxic‐shock syndrome. Due to its lethal nature, all vaginal cultures positive for GAS during pregnancy should be considered a true infection and be treated appropriately with antibiotics. Invasive GAS usually occurs within 2‐3 days of delivery and is characterized by high‐grade fever, chills, and minimal to no abdominal or uterine tenderness.^6^ There may be scant, nonpurulent lochia. Although most GAS infections are associated with bacteremia without focus, endometritis remains one of the top findings of invasive GAS.^7^

Damage to the cardiac tissue, as seen in our patient, is a defining feature of septic shock. Typically, the signs of cardiac insult present as soon as 48 hours after sepsis onset. The massive release of systemic cytokines, including tissue necrosis factor‐alpha (TNF‐alpha), seen in sepsis results in myocardial dysfunction and necrosis.^8^ Pregnancy causes many changes in cardiac function but left ventricular function and ejection fraction are usually unchanged. The use of milrinone provided inotropic support and reduced strain on the heart by decreasing afterload. IVIG has been recommended as additional therapy by promoting an anti‐inflammatory response and neutralizing toxins.^9^ Evidence of this offering additional benefit to GAS TSS is lacking statistical significance in clinical trials.^10^ Laboratory evidence in favor of the use of IVIG in STSS includes the presence of neutralizing antibodies to streptococcal superantigen toxins in IVIG. In addition, plasma from patients with STSS who have been treated with IVIG can inhibit superantigen‐induced T‐cell proliferation and production of cytokines in vitro. IVIG has multiple other anti‐inflammatory properties, and the specific mechanism underlying its potential beneficial effect in STSS is unknown. Much of the known use of IVIG is on patients with viral diseases. The decision to use IVIG for this patient was made and rather or not it provided benefit cannot be concluded from this one case alone but here we describe its use with a positive outcome. The patient was discharged with lisinopril and carvedilol to maintain the reduction in cardiac workload and prevent cardiac remodeling. The cardiac damage sustained has been shown to be fully reversible if treated promptly.^11^

Standard treatment of GAS TSS in pregnancy includes aggressive fluid management, vasopressors, and inotropes to maintain adequate blood pressure and perfusion, antibiotics, and blood products as needed. Penicillin remains the drug of choice for GAS infection.^12^ Because of its potent inhibitory effect on toxin production and additive antimicrobial activity, clindamycin is recommended for severe invasive GAS infection.^13^ The combination of penicillin and clindamycin has been associated with improved outcomes in severe invasive GAS cases. When GAS is found to have colonized the uterus, emergent hysterectomy is recommended to remove the source of infection. Given the young age of the patient, the risks and benefits of this procedure were weighed carefully prior to the operation. Examination during exploratory laparotomy revealed significant damage to the uterus and indicated the need for emergent hysterectomy. The patient's full recovery was facilitated by the staff's ability to provide quick and appropriate management. However, better outcomes could have been achieved had treatment been initiated at an early timeframe. Presented here was a detailed case of GAS TSS and its complications in an adolescent. The goal of this paper was to improve outcomes and avoid serious complications for this vulnerable population by assisting medical professionals in recognizing and treating this rare condition efficiently.

## CONFLICT OF INTEREST

None declared.

## AUTHOR CONTRIBUTION

Samuel Cortez Granados, MD (First Author) was a senior resident in pediatrics and creator of final draft, figures, and tables. Jonathan Batsch (4th‐year medical student) was a co‐writer of the discussion section. Asona Lui, PhD was a co‐writer of clinical case. Jacob Hessman, MD was a senior resident in pediatrics and co‐writer of discussion section. Nika Gloyeske, MD was a provider of pathology sample photographs. Apurva Panchal, MD was a advisor.

## Supporting information

Table S1
