# Supplementary material for: A rare case of group A streptococcal toxic‐shock syndrome in a postpartum adolescent leading to multi‐organ failure
Source: Clin Case Rep. 2020 Mar 16;8(5):793–7. doi: 10.1002/ccr3.2799 (PMC7250980; doi:10.1002/ccr3.2799)
Supplement: Supplementary file 1 — Table S1 [file CCR3-8-793-s001.docx]

Table S1. Summary table listing vasopressors and dosage used during admission.

| Medication | Day of admission | | | | | | | |
| --- | --- | --- | --- | --- | --- | --- | --- | --- |
|  | 0 | 1 | 2 | 3 | 4 | 5 | 6 | Dose* |
| Dexmedetomidine | 0.5 mcg/kg/hr | 0.5 mcg/kg/hr | 0.8 mcg/kg/hr | 1.0 mcg/kg/hr | 1.0 mcg/kg/hr | 0.4 mcg/kg/hr | 0.0 mcg/kg/hr |  |
| Epinephrine | 0.14 mcg/kg/min | 0.05 mcg/kg/min | 0.05 mcg/kg/min | 0.03 mcg/kg/min | 0.01 mcg/kg/min | 0.03 mcg/kg/min | 0.0 mcg/kg/min |  |
| Fentanyl | 75 mcg/hr | 0.0 mcg/hr | 100 mcg/hr | 100 mcg/hr | 50 mcg/hr | 0.0 mcg/hr | 0.0 mcg/hr |  |
| Milrinone | 0.0 mcg/kg/min | 0.0 mcg/kg/min | 0.25 mcg/kg/min | 0.35 mcg/kg/min | 0.25 mcg/kg/min | 0.25 mcg/kg/min | 0.0 mcg/kg/min |  |
| Norepinephrine | 0.14 mcg/kg/min | 0.22 mcg/kg/min | 0.0 mcg/kg/min | 0.0 mcg/kg/min | 0.0 mcg/kg/min | 0.0 mcg/kg/min | 0.0 mcg/kg/min |  |
| Phenylephrine | 150 mcg/kg/min | 0.0 mcg/kg/min | 0.0 mcg/kg/min | 0.0 mcg/kg/min | 0.0 mcg/kg/min | 0.0 mcg/kg/min | 0.0 mcg/kg/min |  |
| Vasopressin | 0.4 u/hr | 0.8 u/hr | 0.4 u/hr | 0.2 u/hr | 0.0 u/hr | 0.0 u/hr | 0.0 u/hr |  |
| Hydrocortisone* | 100 mg | 50 mg | 50 mg | 50 mg | 50 mg | 50 mg | 1. mg |  |
